# Treatment outcomes in HIV-infected adolescents attending a community-based antiretroviral therapy clinic in South Africa

**DOI:** 10.1186/1471-2334-12-21

**Published:** 2012-01-25

**Authors:** Mweete D Nglazi, Katharina Kranzer, Pearl Holele, Richard Kaplan, Daniella Mark, Heather Jaspan, Stephen D Lawn, Robin Wood, Linda-Gail Bekker

**Affiliations:** 1 The Desmond Tutu HIV Centre, Institute for Infectious Disease and Molecular Medicine, and the Department of Medicine, Faculty of Health Sciences, University of Cape Town, Anzio Road, Observatory, 7925 Cape Town, South Africa; 2 International Union against Tuberculosis and Lung Disease, Paris, France; 3 Department of Clinical Research, Faculty of Infectious and Tropical Diseases, London School of Hygiene and Tropical Medicine, London, UK; 4 Department of Health, Provincial Government of the Western Cape, Cape Town, South Africa; 5 Department of Pediatrics, Seattle Children's Hospital, University of Washington, Seattle, WA, USA

**Keywords:** antiretroviral, adolescents, outcomes, mortality, virological failure, Africa

## Abstract

**Background:**

Very few data are available on treatment outcomes of adolescents living with HIV infection (whether perinatally acquired or sexually acquired) in sub-Saharan Africa. The present study therefore compared the treatment outcomes in adolescents with those of young adults at a public sector community-based ART programme in Cape Town, South Africa.

**Methods:**

Treatment outcomes of adolescents (9-19 years) were compared with those of young adults (20-28 years), enrolled in a prospective cohort between September 2002 and June 2009. Kaplan-Meier estimates and Cox proportional hazard models were used to assess outcomes and determine associations with age, while adjusting for potential confounders. The treatment outcomes were mortality, loss to follow-up (LTFU), immunological response, virological suppression and virological failure.

**Results:**

883 patients, including 65 adolescents (47 perinatally infected and 17 sexually infected) and 818 young adults, received ART. There was no difference in median baseline CD4 cell count between adolescents and young adults (133.5 vs 116 cells/μL; *p *= 0.31). Overall mortality rates in adolescents and young adults were 1.2 (0.3-4.8) and 3.1 (2.4-3.9) deaths per 100 person-years, respectively. Adolescents had lower rates of virological suppression (< 400 copies/mL) at 48 weeks (27.3% vs 63.1%; *p *< 0.001). Despite this, however, the median change in CD4 count from baseline at 48 weeks of ART was significantly greater for adolescents than young adults (373 vs 187 cells/μL; *p *= 0.0001). Treatment failure rates were 8.2 (4.6-14.4) and 5.0 (4.1-6.1) per 100 person-years in the two groups. In multivariate analyses, there was no significant difference in LTFU and mortality between age groups but increased risk in virological failure [AHR 2.06 (95% CI 1.11-3.81; *p *= 0.002)] in adolescents.

**Conclusions:**

Despite lower virological suppression rates and higher rates of virological failure, immunological responses were nevertheless greater in adolescents than young adults whereas rates of mortality and LTFU were similar. Further studies to determine the reasons for poorer virological outcomes are needed.

## Background

As the HIV epidemic matures, survival of children with perinatally acquired HIV infection into adolescence is increasingly being documented in sub-Saharan African countries [1,2]. In addition, the burden of HIV in the adolescent patient population in the region is also due to sexual transmission, with adolescents and young adults being particularly vulnerable to this mode of infection [3]. HIV care and treatment services in the region need to adapt to adequately meet the specific needs of this expanding disease burden among adolescents.

Adolescence is a complex development phase of marked psychosocial, behavioural, physiological and cognitive changes [1,4] that exacerbate the challenges of an HIV-positive status and the requirement to adhere to a structured treatment regimen. It is a time when there is hyperawareness of physical appearance [5-7] and also a time of experimentation, risk-taking and significant peer influence with a need to assert an individual identity that is distinct from caregivers [8,9]. The phase is often associated with deviations from expected or prescribed behaviour [9]. These factors may complicate adolescents' transition toward taking responsibility for managing their illness, ART adherence and clinic appointment attendance. Adolescents have been found to have poor adherence to antiretroviral therapy [10-12], with one study showing a decrease in adherence as children moved into adolescence [13]. In addition, low levels of virological suppression [12,14-17], increased risk of virological failure [17], loss to follow-up (LTFU) [15,16] and death have all been described [17].

These studies are almost exclusively from North America and none have directly compared adolescents with young adults. Sub-Saharan Africa accounts for almost 67% of all people living with HIV/AIDS [18] and yet only a handful of studies in the region have investigated outcomes on ART in the emerging group of HIV-infected adolescents [19-21]. We therefore sought to determine treatment outcomes among adolescents (predominantly perinattally infected) attending a large community-based ART service in Gugulethu Township, Cape Town, South Africa, and compared these with young adults attending the same clinic.

## Methods

### Setting

The ART service described here is based in a poor peri-urban settlement of Cape Town, South Africa. ART has been provided for free to the participants since 2002. Clinical and programmatic characteristics of this cohort have been described elsewhere in detail [22-26]. In brief, first-line ART was administered for age-specific severe immunosuppression associated with AIDS diagnosis; or a blood CD4 cell count < 200 cells per microliter for adults and adolescents, according to the 2004 South African national guidelines [27]. The first-line regimens for all ages consisted of two nucleoside reverse transcriptase inhibitors (NRTI) and a non-nucleoside reverse transcriptase inhibitor (NNRTI). During the period of analysis, adolescents and adults were initiated on stavudine (d4T), lamivudine (3TC) and efavirenz (EFV) or nevirapine (NVP), unless contraindicated. All CD4 count and viral load tests were performed without cost to the patients by the Toga Laboratory "Togatainer" that was located onsite. CD4 cell counts using flow cytometry (FACSCount™, Becton Dickinson, Franklin Lakes, NJ, USA) and plasma HIV-RNA levels using the branch DNA hybridisation technique (Versant™ HIV-1 RNA 3.0 branched chain DNA assay, Bayer HealthCare, Leverkusen, Germany; detection range of 50-500 000 copies/mL) were measured prior to commencing ART and then at weeks 0, 4, 8, and 16 on treatment, and with regular 16-weekly follow-up thereafter.

Due to the escalating numbers of perinatally infected children on ART reaching adolescence an adolescent- centered service was introduced in April 2008 to address the needs of this emerging group. This service integrated adherence monitoring and support; peer support groups; counselling education delivered by well trained and sensitised staff and psychosocial support services for adolescents and caregivers within a non-judgemental environment.

In this study, the majority [16/19 (84.2%)] of the adolescents were referred from the Red Cross War Memorial Children Hospital, Cape Town, and other referral hospitals, while the rest were from primary level or private facilities.

### Data collection

Data on clinical variables, outcomes, treatment regimens and laboratory records were routinely collected. A cohort study of HIV-infected adolescents and young adults on ART between September 2002 and June 2009 was done. Treatment outcomes were censored on 30 June 2010. Ethical clearance was obtained from the Research Ethics Committee of the University of Cape Town. Written informed consent to have data collected anonymously for research purposes was obtained from all adult patient 18 years and older and caregiver consent was obtained for all adolescent patients under 18 years.

### Definitions

#### Age group

In the present study the definitions used for adolescents (age 9-19 years) and young adults (20-28 years) were similar to that used in the literature [19,28]. Data analysed in these two age groups were based on age at ART initiation and patients were not switched between age groups during follow-up.

#### Outcomes

LTFU referred to patients that had failed to attend the clinic for ≥ 12 weeks and were not known to have died or to have been transferred out to another facility.

Mortality referred to all-cause deaths notified from any source.

Median change in CD4 counts were calculated by subtracting the CD4 count measurements at each time point after ART initiation from the baseline measurement.

Virological suppression referred to patients achieving a viral load < 400 copies/mL

Virological failure referred to patients who had initially achieved virological suppression (< 400 copies/mL) with two subsequent viral load measurements > 1000 copies/mL.

### Statistical analyses

Descriptive statistics were used to characterize the demographic, clinical and laboratory variables (at baseline and/or specific time points after ART initiation). Comparisons between adolescent and young adult status were examined using chi-square tests for proportions (replaced by Fishers exact test for sparse data), Wilcoxon rank-sum tests and Kruskal Wallis test (for medians). Kaplan-Meier estimates were used to describe time-to-event distributions for the following outcomes: mortality, LTFU and virological failure. The endpoint was the time from ART initiation to one of the following events: death, loss to follow-up and second consecutive viral load > 1000 copies/mL. Censoring occurred at the date of death, date of transfer to another ART facility, date of loss to follow-up or study end (i.e. 30 June 2010), whichever occurred first. Multivariate Cox proportional hazard models were used to estimate hazard ratios adjusting for potential confounders defined a priori using the following variables: sex, baseline CD4 cell count and baseline viral load. The proportional hazards assumption has been checked graphically using a log-log plot and the Schoenfeld residuals (tests and graphs). Analyses were conducted among all patients (treatment-naïve and experienced) with sub-analyses among treatment-naïve patients where appropriate. STATA statistical software, version 11.0 was used for analyses (STATA Corporation, College Station, Texas, USA).

## Results

### Baseline characteristics

A total of 883 patients aged 9-28 years had received ART at this service by end of June 2009. Of these, 65 were adolescents and 818 were young adults. Nineteen (29.2%) adolescents and 103 (12.6%) young adults had transferred into care already on ART. Adolescents had a median age of 11.5 years [interquartile range (IQR) 10.0-17.3] with 44 (67.7%) in early (9-14 years), 8 (12.3%) in middle (15-17 years) and 13 (20.0%) in late (18-19 years) adolescence. The majority (N = 47, 72.3%) of adolescents had perinatally acquired HIV infection, while 26.2% (N = 17) of them had sexually acquired infection. A significantly higher proportion of young adults than adolescents were female [66.2% (43/65) vs. 86.6% (708/818); *p *< 0.0001]. Based on this ratio and age, our assumption is that most if not all of the young adult population had non-perinatally acquired HIV infection. Immunodeficiency was advanced in both adolescents and young adults, as reflected by baseline CD4 cell counts (Table 1). Median baseline CD4 cell count did not differ between adolescents and young adults (133.5 vs 116 cells/μL; *p *= 0.31). The majority of adolescents and young adults received NNRTI-based ART at cohort entry [98.5% (64/65) and 98.9% (809/818), respectively] (Table 1). There was no difference in duration of follow-up in the ART cohort in adolescents compared with young adults [median 34.6 months; IQR of 17.8-48.1 vs. 31.1 months (IQR: 15.3-49.2); *p *= 0.59] (results not shown here).

**Table 1:** Baseline Characteristics of HIV-infected adolescents (9-19 years) and young adults (20-28 years) at a South African antiretroviral treatment service

Characteristics	Adolescents N = 65	Young adults N = 818	p-value
Transferred-in patients, n (%)	19 (29.2)	103 (12.6)	

Female, n (%)	43 (66.2)	708 (86.6)	< 0.0001

CD4† (cells/μL), median (IQR)	133.5 (41-198)	116 (56-170)	0.31

Baseline viral load** (copies/ml), log_10 _median IQR)	4.8 (4.5-5.2)	4.8 (4.4-5.3)	0.74

ART regimen at cohort entry, n (%)			

EFV-based	53 (81.5)	413 (50.5)	< 0.001

NVP-based	11 (16.9)	396 (48.4)	

PI-based	1 (1.5)	9 (1.1)	

†Data are available for adolescents 89.2%, young adults 88.7%

**Data are available for adolescents 80.0%, young adults 81.0%

### Programme losses (mortality and lost to follow-up)

Overall, the mortality rate was 2.9 per 100 person-years [95% confidence interval (CI) 2.3-3.7] and the LTFU rate was 10.0 per 100 person-years (95% CI 8.8-11.4). Rates of mortality and LTFU disaggregated by age group are described in Table 2. There was no significant difference in mortality and LTFU in unadjusted and adjusted analysis (Table 2).

**Table 2:** Programme losses (mortality and lost to follow-up (LTFU) in all adolescents and perinatally infected adolescents (9-19 years) compared with that of young adults (20-28 years)

	Mortality			LTFU		
	
Age group	Rates (per 100 PYS)	AHR*	p-value	Rates (per 100 PYS)	AHR*	p-value
All adolescents	1.2 (0.3-4.8)	0.56 (0.13-2.32)	0.42	7.2 (4.1-12.6)	0.74 (0.41-1.34)	0.33

Perinatally infected adolescents	0.8 (0.1-5.5)	0.39 (0.05-2.86)	0.35	3.9 (1.6-9.4)	0.39 (0.16-0.96)	0.04

Young adults (ref)	3.1 (2.4-3.9)	1.00	-	10.2 (9.0-11.7)	1.00	-

*PYS *person-years; *UHR *unadjusted hazard ratio; *AHR *adjusted hazard ratio

*adjusted for gender, baseline CD4, baseline viral load

In a sub-analysis comparing perinatally infected adolescents and young adults, there was no significant difference in mortality in unadjusted and adjusted analyses; but, there was a significantly lower risk of LTFU in perinatally infected adolescents [AHR 0.39 (95% CI 0.16-0.96; *p *= 0.04)] (Table 2).

### Immunological and virological response

The immunological response to ART in all adolescents was compared with that of young adults. Patients who had transferred into care on ART were excluded in this analysis since baseline CD4 counts were not known. The median change in CD4 count from baseline at the various time points were significantly higher for all adolescents than in young adults (Table 3). At 48 weeks, the median change was higher for all adolescents than young adults (373 vs 187 cells/μL; *p *= 0.0001). When we compared perinatally infected adolescents and young adults, a similar pattern was observed with these adolescents having significantly higher median changes in CD4 count (Table 3).

**Table 3:** Immunological and virological response to ART among all ART-naïve adolescents and perinatally infected adolescents (9-19 years) compared with that of young adults (20-28 years)

Characteristics	All ART-naïve adolescents N = 64	ART-naïve young adults N = 715	P-value†	ART-naïve perinatally-infected adolescents N = 31	P-value†
Baseline CD4 (cells/μL), median (IQR)	139 (62-198)	117 (59-170)	0.15	170 (78-228)	0.026

CD4 (cells/μL), median (IQR) change from baseline					

16 weeks	220 (133.5-314)	121 (67-199)	0.0001	188.5 (126-322)	0.0004

32 weeks	195 (136-439)	155 (97-238)	0.0008	256 (172-487)	0.0002

48 weeks	373 (255-518)	187 (118-285)	0.0001	374 (242-631)	0.0002

Virological suppression*, n(%)					

16 weeks	36 (97.3)	490 (89.6)	0.13	27 (100.0)	0.077

32 weeks	12 (37.5)	331 (75.1)	< 0.001	7 (28.0)	< 0.001

48 weeks	9 (27.3)	270 (63.1)	< 0.001	6 (24.0)	< 0.001

†The p values were compared with young adults and calculated using Wilcoxon rank-sum test for continuous variables and fishers exact test for binary variables.

*The denominators at 16 weeks for all ART-naïve adolescents, perinatally infected adolescents and young adults were 37, 27 and 547 respectively; at 32 weeks was 32, 25 and 440 respectively and at 48 weeks was 33, 25 and 427 respectively.

We then compared the virological response to ART in adolescents to that of young adults. Patients who had transferred in to care on ART were excluded in this analysis since baseline HIV RNA viral loads were not known. We found the proportion of surviving adolescents achieving virological suppression (< 400 copies/mL) was lower than young adults, and the difference was significant after 48 weeks of ART [27.3% (9/33) vs 63.1% (270/428), *p *< 0.001] (Table 3). When we compared perinatally infected adolescents and young adults, a similar pattern was observed (Table 3).

### Virological failure

**Figure 1:**
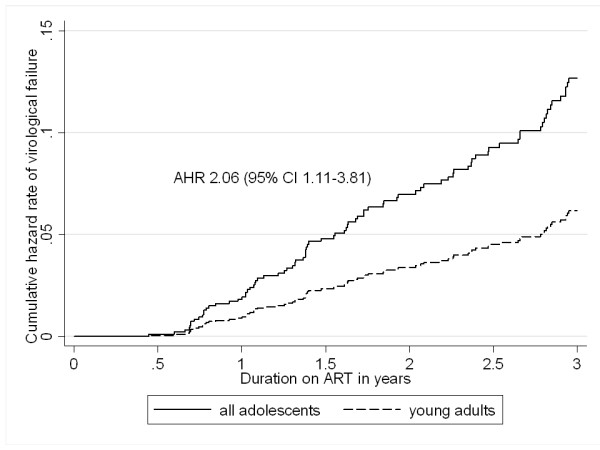
**Adjusted Cox proportional hazard regression model for virological failure among adolescents and young adults**. Virological failure was defined as 2 consecutive viral loads above 1000 copies/mL and adjustment was made for age, baseline CD4 count and viral load.

The rate of virological failure among all patients was 5.2 (95% CI 4.3-6.3) per 100 person-years. The rate of virological failure in adolescents and young adults were 8.2 (95% CI 4.6-14.4) and 5.0 (95% CI 4.1-6.1) per 100 person-years, respectively. There was a significantly higher rate of virological failure in adolescents compared to young adults [AHR 2.06 (95% CI 1.11-3.81; *p *= 0.02)] (Figure 1). However, this association was weakened [AHR 1.51 (95% CI 0.68-3.33); *p *= 0.31] in a sub-analysis comparing perinatally infected adolescents and young adults.

## Discussion

In this study, adolescents were less likely to attain virological suppression and had higher rates of virological failure compared to young adults. Despite the poorer virological outcomes, immunological responses were nevertheless greater in adolescents than young adults. There was no significant difference in mortality and LTFU rates between the two groups. In sub-analyses comparing perinatally infected adolescents with young adults, broadly similar results were observed with regards to mortality, immunological and virological response to ART. However, the association between adolescent status and the higher rates of virological failure was weakened.

Our findings of a lower rate of virological suppression and increased rate of virological failure in this public health sector study are cause for concern considering the long-term antiretroviral need these adolescents have. These results are similar to those of a recent multi-site study in the private health sector of Southern Africa by Nachega et al. [19]; but differ slightly from those from another public health sector study from a peri-urban settlement (Khayelitsha, Cape Town) by van Cutsem et al. [20] which observed that virological outcomes were worse for youth (20-24 years) when compared to those in the adolescents (10-19 years) and adults (≥ 25 years).

Virological suppression requires effective ART. This implies the correct dose of appropriate ART being consistently and correctly taken. Adolescents whilst undergoing a range of psychosocial developments are also growing physically. Consequently, it may be important to review the appropriateness of ART dosing as children transition from paediatric doses into adult doses [29]. This may be particularly relevant in younger adolescents. Adequate options for paediatric appropriate antiretroviral drugs may also play a role in the outcomes in adolescents. Often choices are limited and formulations are inappropriate. Adequate adherence to medication may on the other hand, be an important factor in older adolescents and youth where all of the psychosocial factors described here [1,4] may play a role in an individual's ability or willingness to be adherent. In many low and middle income countries, where prevention of mother-to-child transmission (PMTCT) strategies have include monotherapy with NVP, the impact on non-nucleoside based first-line regimens is also for consideration. The impact of maternal PMTCT, and in particular the impact of single dose NVP on this cohort is unlikely since the youngest adolescents described here were born in 2001 before the clinic and provincial- and country-wide roll-out of PMTCT [30].

Despite the poorer virological outcomes, we observed that the median change in CD4 count from baseline was higher for all adolescents and perinatally infected adolescents than young adults, suggesting that younger and more robust immune systems occur in adolescents. A study conducted in the United States and Peurto Rico conducted by Flynn et al. [16] also report improved CD4 cell count measurements in adolescents infected via high- risk behaviours after 3 years of follow-up in subjects with sustained undetectable viral loads. In contrast, a Southern African observational cohort study conducted by Nachega et al. [19] reported that adolescents (10-19 years) were less likely to experience long-term immune recovery than young adults (20-30 years). These contrasting findings may be due to differences in adolescent population between studies in terms of age and mode of infection; our adolescent population consisted of younger adolescents with predominantly perinatally acquired infection who had relatively better responses to ART compared to the older adolescents (median age, 16.4 years; 73% female) in the Nachega cohort (presumably with sexually acquired infection on the basis of age and sex ratio).

Our findings did not show a significant difference in mortality and LTFU rates between the groups. Literature on mortality and LTFU in adolescents is scant. The absence of any statistically significant difference in mortality and LTFU between adolescents and young adults in the present study is similar to reports from a recent study conducted in a public health sector ART cohort from Uganda by Bakanda et al. [21]. Whilst there was no significant difference between mortality and LTFU, there were still some losses (death and LTFU) to the program that need corrective interventions. Site- and patient-related factors, such as lack of compensation (for participants), lower caregiver education level, older age and higher viral load, have all been associated with increased LTFU in HIV infected children and adolescents in a multi-site cohort study conducted across Puerto Rico and the United States by Williams et al. [31]. This study does not ascertain what factors are important in these two age groups and may be quite different. Further research on factors associated with and the reasons for both LTFU and mortality in adolescents are required.

Compared to analyses using the complete group of adolescents, sub-analyses restricted to perinatally infected adolescents showed a weaker association between adolescent status and higher rates of virological failure compared to young adults. Collectively, these data may suggest that the higher rates of virological failure among adolescents were more strongly associated with those with sexually acquired infection. In addition, perinatally infected adolescents had a significantly lower risk of LTFU, which may be attributable to the fact that such individuals were more likely to attend the treatment facility with the support of parents, family and guardians than the older adolescents with sexually acquired infection. This hypothesis is supported by a recent qualitative study conducted among perinatally infected adolescents aged 7-15 years in Cape Town that found they viewed HIV as physically and emotionally painful, but strong family and friend support systems were viewed as positive aspects of their lives [4]. Thus, differences in outcomes between the two sub-group of adolescents may be related to a range of medical, physiological, psychosocial, psychosexual and neuro-cognitive issues [1,29,32].

The recent modification of services in this clinic to include a dedicated adolescent service may or may not be influencing adolescent outcomes since the cohort included adolescents from before as well as after the introduction of new adolescent services. Recommendations from other medical fields where adolescents may have chronic illnesses requiring ongoing medication adherence are that adolescent friendly and specific services are more effective [33-37]. With longer follow up, the programmatic impact of this dedicated adolescent service may be assessed. This study is unusual in that it explores treatment outcomes among all adolescents, including those transferred in on treatment and compares this to young adults. This gives insights into the overall programmatic outcomes of the adolescent population in this public sector ART clinic in Cape Town, especially useful given the traffic between clinics and within services with the realisation of greater treatment accessibility in Southern Africa. Although the study was conducted in a relatively large community-based clinic, and the numbers of adolescents in public sector ART programs in Southern Africa are accumulating, the numbers of adolescents involved in this cohort is still small, which is a limitation of this study. In addition, other limitations include the lack of data on adherence, viral resistance mutations, side-effects to therapy, all of which will require investigation in future studies. The data presented here does however indicate that this group in community-based ART programs should be evaluated separately to the adult population, and where possible perinatally infected adolescents and sexually infected adolescents may have different outcomes and warrant separate evaluation; and similarly adolescent specific corrective interventions should be implemented. Therefore, further research into understanding the reasons for LTFU, mortality, immunological and virological outcomes in this group in whom lifelong antiviral suppression is required.

## Conclusions

In summary, despite lower virological suppression rates and higher rates of virological failure, immunological responses were nevertheless greater in adolescents than young adults whereas rates of mortality and LTFU were similar. Whilst this clinic has taken the steps to introduce adolescent specific services, this has only been active since 2008 and the future assessment of such services and other interventions on mortality, LTFU, immunological and virological outcomes in adolescents are anticipated.

## Competing interests

The authors declare that they have no competing interests.

## Authors' contributions

MDN, KK, LGB designed the study. MDN and PH collected the data and MDN did the analyses with input from KK and LGB. PH, RW and LGB developed the research infrastructure and implemented the adolescent clinic. MN and LGB wrote the paper with input from KK, PH, RK, DM, HJ, SDL, and RW. All authors read and approved the final manuscript.

## Pre-publication history

The pre-publication history for this paper can be accessed here:

http://www.biomedcentral.com/1471-2334/12/21/prepub
